# Diagnostics and treatment of ovarian cancer in the era of precision medicine - opportunities and challenges

**DOI:** 10.3389/fonc.2023.1227657

**Published:** 2023-09-06

**Authors:** Patrycja Aleksandra Bukłaho, Joanna Kiśluk, Jacek Nikliński

**Affiliations:** ^1^ Department of Clinical Molecular Biology, Medical University of Bialystok, Bialystok, Poland; ^2^ Doctoral School, Medical University of Bialystok, Bialystok, Poland

**Keywords:** ovarian cancer, HGSOC, targeted therapy, cancer screening, HBOC (hereditary breast and ovarian cancer), cancer precision medicine

## Abstract

Due to predictions of increasing incidences and deaths from ovarian cancer, this neoplasm is a challenge for modern health care. The advent of NGS technology has made it possible to understand the molecular characteristics of many cancers, including ovarian cancer. The data obtained in research became the basis for the development of molecularly targeted therapies thus leading to the entry of NGS analysis into the diagnostic process of oncological patients. This review presents targeted therapies currently in preclinical or clinical trials, whose promising results offer hope for their use in clinical practice in the future. As more therapeutic options emerge, it will be necessary to modify molecular diagnostic regimens to select the best treatment for a given patient. New biomarkers are needed to predict the success of planned therapy. An important aspect of public health is molecular testing in women with a familial predisposition to ovarian cancer enabling patients to be included in prevention programs. NGS technology, despite its high throughput, poses many challenges, from the quality of the diagnostic material used for testing to the interpretation of results and classification of sequence variants. The article highlights the role of molecular testing in ongoing research and also its role in the diagnostic and therapeutic process in the era of personalized medicine. The spread of genetic testing in high-risk groups, the introduction of more targeted therapies and also the possibility of agnostic therapies could significantly improve the health situation for many women worldwide.

## 1 Introduction

Ovarian cancer (OC) is diagnosed in a large number of women around the world annually - in 2020, 313,959 new cases were reported - significantly reducing their comfort level and also taking the lives of many of them (207,252 deaths) (1, 2). OC ranks seventh in cancer incidence and is fifth leading cause of cancer-related death in women worldwide (1), in addition is considered to be the most lethal gynecological cancer (3). Statistical studies have shown a general downward trend in the incidence and mortality of ovarian cancer over the past decade linked, no doubt, to advances in medicine. However, the significant growth in incidence in younger women is worrisome (2).

It is known that the increased risk of developing ovarian cancer is related to genetic predisposition and family history (Hereditary Ovarian Cancer - HOC). Hereditary cancers, i.e. those caused by inherited mutations in genes that predispose to cancer development, account for as many as 20-25% of epithelial ovarian cancer cases (4). It is worth mentioning that breast and ovarian cancers show some common features at the molecular level (5). The distinct disease syndrome is Hereditary Breast and Ovarian Cancer (HBOC) in which the risk of breast and ovarian cancer is higher than in the general population (6). Breast cancer cases due to HBOC are estimated to account for 2 to 7%; for ovarian cancer, the range is 15 to 18% (4).

Whether spontaneously occurring or associated with a genetic predisposition, OC represents a major global problem and a challenge of modern medicine. Undoubtedly, the development of next-generation sequencing (NGS) technology has contributed to a better knowledge and understanding of cancer biology and, moreover, in the detection of high-risk groups (7). This is crucial in the early diagnosis of cancer and also in the selection of targeted therapy (8). NGS panels approved for use in patient diagnosis allow assessment of both somatic and germline variants, as well as single or global genomic changes (7). Such broad capabilities of NGS technology make it widely used in scientific research and patient diagnostics, including oncology, and an important step towards personalized medicine. However, many factors influence the success and improvement of NGS research, such as sequencing chemistry, sequencing technology, bioinformatics and data analysis (9).

## 2 Molecular pathogenesis of ovarian cancer

High-grade serous ovarian cancer (HGSOC) is the most common histological type of ovarian cancer and accounts for about 75% of ovarian cancers of epithelial origin (10). Despite the common histological features identified by pathologists, HGSOC tumors have significant differences in the clinical course of the disease, including response to treatment (11). HGSOCs exhibit significant heterogeneity due to differences at the molecular level (12).

The *TP53* somatic mutation occurs as the first molecular alteration involved in HGSOC carcinogenesis and is referred to as a driver mutation. This condition was identified in 96% of HGSOC cases (3). The protein encoded by *TP53*, called p53, is a transcription factor that activates genes engaged in DNA repair, cell cycle and apoptosis after nonreversible DNA damage (13). Loss of p53 function promotes tumorigenesis, but does not itself cause a malignant phenotype, and at least one more genotoxic event is required. Most frequently, this is a *BRCA1/2* mutation. BRCA1/2 can lose its functionality through somatic mutation, germline mutation or through promoter hypermethylation, which belongs to epigenetic modifications. It is worth noting that mutations are mutually exclusive with epigenetic silencing of the *BRCA1/2* gene (3). BRCA1 and BRCA2 proteins are involved in DNA repair through homologous recombination (homologous recombination repair (HRR)) (14). Defects in HRR are estimated to occur in 50% of HGSOC cases. Besides changes in *BRCA1/2*, it can be caused by mutations in *ATM*, *BRIP1*, *CHEK2*, *NBN*, *PALB2*, *RAD51B (* 3, 14). However, an HGSOC tumor can acquire genomic instability through disruption in another pathway. *BRCA1/2* loss of function is mutually exclusive with *CCNE1* amplification and *RB1* inactivation. Both *CCNE1* and *RB1* are involved in the Rb cell cycle regulation pathway (3). The protein cyclin E1 forms a complex with cyclin-dependent kinase 2 (CDK2) to promote cell cycle progression from G1 to S phase (15). Cyclin E1 expression relies on E2F transcription factors that are bound to retinoblastoma protein (Rb) (16). Amplification of *CCNE1* and deletion of *RB1* hasten the cell cycle, leading to defective S-phase progression and an elevated number of chromosome breaks, resulting in genomic instability. *CCNE1* amplification appears in about 20% of HGSOC cases while *RB1* inactivation occurs in 10% of HGSOC (3).

## 3 Targeted treatment options – available now and under study

Currently, the standard of care for patients with stage II-IV ovarian cancer is platinum-based chemotherapy and surgical cytoreduction (16). Ovarian tumors with HRD are characterized by higher sensitivity to platinum and poly(ADP-ribose) polymerase inhibitor (PARPi) therapies. When *RB1* function is lost, the tumor is also sensitive to platinum based therapies. In contrast, ovarian cancer with *CCNE1* overexpression shows greater resistance to platinum compound therapies (17). Identifying the pathogenesis of the tumor at the molecular level allows the choice of targeted therapy.

### 3.1 Tumors with HRD including loss of *BRCA1/2* function

#### 3.1.1 PARP inhibitors monotherapy

For the treatment of breast and ovarian cancer, the Food and Drug Administration (FDA) has approved several PARPi. These drugs are effective for cancer patients with mutated *BRCA1/2* and for treating recurrent platinum-sensitive ovarian cancer (18, 19). A meta-analysis by Staropoli et al. of 1,839 epithelial ovarian cancer patients showed a statistically significant benefit from PARPi. A prolongation of survival time was confirmed, with no significant differences in progression-free survival (PFS) for the individual pharmaceuticals in this group (20). PARPi acts through synthetic lethality with loss of BRCA1/2 functionality. This occurs due to the destabilization of replication forks by PARPi, homologous recombination does not function properly in these cells and consequently DNA double-strand breaks (DSBs) occur, which cause cell death (21, 22). Initially, PARPi was used only in patients with an identified *BRCA1/2* mutation. However, the treatment is known to be effective for homologous recombination deficiency (HRD), which can be also caused by mutations in other genes. This approach has allowed a significant expansion of the group of patients in whom targeted therapy can be used (23). The use of PARPi in clinical practice has resulted in improved PFS. Despite its high efficacy, not all patients with tumors with mutations in *BRCA1/2* or HRD respond to treatment, and moreover, most patients eventually develop resistance (24).

#### 3.1.2 Combination therapies to improvePARPi efficacy and therapeutic strategiesfor PARPi resistance

Strategy that appears to be very promising in clinical practice is combination therapy involving PARP inhibitors and antiangiogenic drugs. The combination of these medications has been shown to result in a significant prolongation of PFS in both women with newly diagnosed advanced OC and with recurrent disease (25). Combinations with PI3K/AKT (phosphoinositide 3-kinase/protein kinase B), EGFR (epidermal growth factor receptor) and BET (bromodomain and extraterminal domain) inhibitors are proposed as other therapies that can overcome PARPi resistance (24). Noteworthy are the findings of Gasimla et al. The researchers analyzed the molecular profiles of BRCA2-deficient HGSOC tumors resistant to PARPi. They concluded that in these cases *KRAS* amplification is responsible for the resistance. The researchers suggest that inhibition of PLK1 (Polo-Like Kinase 1), which plays a role in regulating cell division, restores PARPi sensitivity (19). Immunogenicity plays an important role in the response to treatment. Kraya et al. showed differences in the immunogenicity of ovarian tumors with mutations in *BRCA1/2* and distinguished two groups: tumors with *PTEN* loss and *BRCA1* promoter hypermethylation, and tumors with wild-type *PTEN*. Cancers in the first group were characterized by significantly reduced CD3+, CD8+, and FOXP3+ T cell composition and higher HRD scores. The researchers suggest that combination therapy involving PARPi and immune checkpoint blockade (ICB) may be effective in patients with *BRCA1/2* deficiency and wild-type *PTEN (* 26). Shein et al. suggest that assessing the expression of immune-related genes may be an effective biomarker for estimating OS in women with ovarian cancer (27). The incorporation of epigenetic regulation into therapy was explored by Pulliam et al. Researchers have shown that inhibition of DNMT1 (an enzyme involved in DNA repair) sensitizes breast and ovarian cancers resistant to PARP inhibitor therapy. Moreover, DNMTi and PARPi therapy is effective regardless of *BRCA* status (18). Gupta et al. reported synergistic effects of histone deacetylase inhibitors (HDACi) and PARPi *in vitro* in ovarian cancer cells with an efficient homologous recombination mechanism. HDACi induces defective DNA repair, leading to an increase in the number of DSBs and, by repressing HR, enhances the action of PARPi. The researchers indicate that this therapy could have significant effects in poor-prognosis tumors with *CCNE1* amplification (28). The challenge is to develop a scheme to classify patients for monotherapy or combination therapy. Necessary for this is the search for biomarkers, especially at the molecular level (25, 26).

Poly(ADP-ribose) glycohydrolase (PARG) inhibitors, similarly to PARPi, destabilize replication forks and induce DNA damage (21). However, PARG inhibitors show activity in *BRCA* wild-type cells, making them an effective alternative for patients with primary or acquired PARPi resistance (29). Successful completion of clinical trials on the efficacy of PARG inhibitors and a deeper understanding of their mechanism of action, as well as finding markers to predict susceptibility to therapy, is essential for the development of a new targeted drug effective in many types of cancer, not limited to ovarian cancer (21, 29).

### 3.2 Tumors with *CCNE1* amplification

*CCNE1* amplification is common in many types of cancer. Tumors with this molecular alteration are characterized by relative resistance to chemotherapy and, in addition, van Wagensveld et al. showed a lower content of immune cells which may result in a worse response of patients to immunotherapy (30). Therefore, finding targeted therapies for this genetic change has become a challenge for many researchers. Along with *CCNE1* amplification, *TP53* mutation is often present. In this group, significantly longer overall survival (OS) was observed in patients who received antiangiogenic therapy (31). One other strategy in cancers with *CCNE1* amplification is CDK2 inhibition. The resulting CDK2-cyclin-E1 complex plays a role in chromosomal instability. Therefore, CDK2 inhibitors could inhibit tumorigenesis. *CCNE1* amplification could be considered a biomarker of susceptibility to CDK2 inhibition. However, the oncogenic effect of cyclin E1 independent of CDK2 should be considered (32). Gallo et al. conducted studies in cell models and showed that *CCNE1* overexpression results in susceptibility to PKMYT1 kinase inhibition. PKMYT1 kinase is a negative regulator of CDK1 and the selective inhibitor developed by the researchers appears to be an effective therapeutic strategy. The drug is intended for oral use, and clinical trials are currently ongoing for use as monotherapy or in combination with gemcitabine (33). High levels of cyclin E1 cause upregulation of the ATR axis, which induces the ATR/Chk1/WEE1 phosphorylation cascade (34). Xu et al. conducted studies on cell lines and xenografts with *CCNE1* overexpression. The researchers showed that this genetic alteration causes sensitivity to WEE1 inhibition and ataxia telangiectasia-associated Rad3 (ATR) inhibition (WEE1i-ATRi). Preclinical studies on a number of such drugs are currently underway and four ATRi and one WEE1i are in phase I - II clinical trials for efficacy in cancer therapy (35, 36). Xu et al. suggest that by combining the two inhibitors, it will be possible to use lower doses of the drug, thereby reducing toxicity. However, the authors emphasize that treatment with WEE1i-ATRi, despite good tolerance in mice, requires *in vivo* studies before use in clinical practice (15).

### 3.3 Tumors with amplification of other genes

In addition to the most common amplification found in HGSOC which is *CCNE1* amplification, somatic copy number alterations (SCNAs) also affect *MYC*, *PIK3CA*, *KRAS* and *TERT* and may also become targets for personalized therapy. Martins et al. show that *MYC* copy number correlates with the *in vitro* response to mTORC1/2 inhibition. Moreover, the researchers showed that activation of the mTOR survival pathway in ovarian tumors with *MYC* amplification is associated with a higher frequency of SCNAs in PI3K pathway genes. The coexistence of these two SCNAs also occurs in squamous cell lung cancer and triple-negative breast cancer (37). Meanwhile, Martins et al. demonstrated the efficacy of combination therapy involving mTOR pathway inhibition and paclitaxel in HGSOC tumors with *MYC* amplification (38). Transcription of the *MYC* oncogene depends on CDK7, CDK12, and CDK13, among others. Zeng et al. propose as a therapeutic strategy the use of THZ-1, which inhibits the aforementioned cyclin-dependent kinases which leads to a decrease in *MYC* expression levels and thus inhibition of tumor growth (39).

### 3.4 Tumors with *RB1* inactivation

*RB1* mutations are found in several cancer types including OC, which are further characterized by resistance to available therapies. Gong et al. tested 36 cell cycle inhibitors, of which the Aurora kinase inhibitors AURKA and AURKB showed the strongest synthetic lethality against small cell lung cancer (SCLC) cell lines. AURKA showed significantly higher selectivity compared to AURKB and was also well tolerated by mice (40). While Oser et al. demonstrated the efficacy of Aurora kinase B on SCLC cell lines with *RB1* loss of function, they also point to good tolerance in experimental animals and suggest the possibility of using it in other types of cancer with this genetic alteration (41).

**Figure 1:**
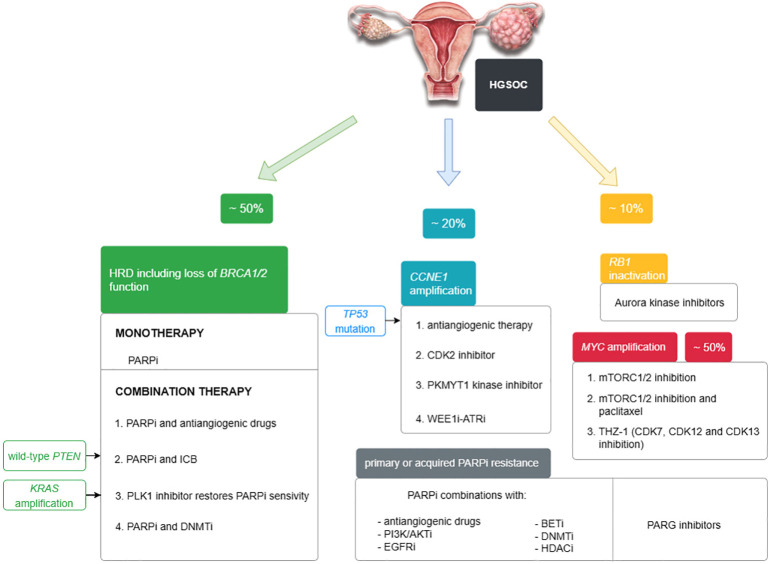
Targeted treatment options for HGSOC approved by the FDA and under study described in the text.

**Figure 1** and **Table 1** summarize the treatment options for women with HGSOC that may be possible in the near future.

**Table 1:** Targeted treatment options in OC available now and under study.

Targeted treatment options in ovarian cancer	
available now (approved by FDA)	under study
• PARPi (poly(ADP-ribose) polymerase inhibitor) • PARPi + antiangiogenic drugs	• PARPi + ICB (immune checkpoint blockade) • PARPi + PI3K/AKTi (phosphoinositide 3-kinase inhibitor/protein kinase B inhibitor) • PARPi + BETi (bromodomain and extraterminal domain inhibitor) • PARPi + PLK1i (Polo-like Kinase 1 inhibitor) • PARPi + DNMTi (DNA methyltransferase inhibitor) • PARPi + HDACi (histone deacetylase inhibitor) • PARGi (poly(ADP-ribose) glycohydrolase inhibitor) • CDK2i (cyclin-dependent kinase 2 inhibitor) • PKMYT1i (Protein Kinase, Membrane Associated Tyrosine/Threonine 1 inhibitior) • WEE1i + ATRi • mTORC1/2i • mTORC1/2i + paclitaxel • CDK7i + CDK12i + CDK13i (cyclin-dependent kinase 7, 12 and 13 inhibitors) • AURKA and AURKB (Aurora kinase inhibitors)

## 4 Hereditary predisposition to ovarian cancer

Most cases of HOC and HBOC are determined by germline mutations in *BRCA1* or *BRCA2 (* 6, 42). It is estimated that about 1 in 400-800 individuals carry the germline pathogenic variant of the *BRCA1* and/or *BRCA2* genes (43). Patients are placed under medical supervision if diagnosed as carrying a *BRCA* mutation. Chemoprevention or risk-reducing surgical treatment: risk‐reducing mastectomy and risk‐reduction salpingo‐oophorectomy may be used to reduce the risk of developing breast and ovarian cancer (44).

As for familial or hereditary ovarian cancer syndromes, they are caused by inherited mutations in tumor suppressor genes. In addition to *BRCA1* and *BRCA2* genes belonging to the Fanconi anemia pathway, mutations in *PALB2*, *ATM*, *RAD51C/D* and *BRIP1* are also known to increase the risk of ovarian cancer. Another important pathway is the mismatched DNA repair pathway and genes such as *MLH1*, *MSH2* and *MSH6 (* 42).

Beyond the increased risk of developing breast and ovarian cancer, individuals in the HBOC group are at increased risk of developing other cancers, such as melanoma, pancreatic cancer and, in the case of men, prostate cancer (6). The risk of developing a specific cancer is related to the type of mutation. Women who have inherited the pathogenic *BRCA1* variant tend to suffer from ovarian cancer at a younger age. In contrast, patients with a mutation in *BRCA2* have a greater predisposition to develop male breast cancer, prostate cancer, pancreatic cancer and melanoma (45). Other HBOC-related genes include *ATM*, *BRIP1*, *CDH1*, *PALB2*, *PTEN*, *RAD51C*, and *TP53 (* 46). Velázquez et al. used a panel of 35 genes to study hereditary cancers. In the case of HBOC, pathogenic variants were noted in the following genes: *BLM*, *BRCA2*, *BRIP1*, *CHEK2*. The researchers underline that by using the expanded panel, they were able to detect pathogenic variants that would not have been detected with routinely used diagnostic panels (47). Resch et al. also demonstrated the benefits of using wider gene panels in their study. Most of the pathogenic variants identified were *BRCA1*, *BRCA2*. However, they also detected variants in *CHEK2* and *RAD51C* and also in *ATM*, *BARD1*, *MUTYCH* and *SMARCA4* which would not have been possible with a standard panel (48).

It should also be taken into account that the etiology of HBOC may be related to constitutional mosaic variants. These variants can involve somatic and also germinal cells, and can therefore be passed onto offspring. In their work, Hidalgo Mayoral et al. described a clinical case of a breast cancer patient in whom they detected a mosaic variant c.9648 + 1G>A in the *BRCA2* gene. The authors point to other cases reported in the literature and emphasize the role of *BRCA1/2* mosaic variants in HBOC. Due to the low allele frequency (VAF), many of these may have been missed in genetic analyses. A separate diagnostic algorithm is needed to detect potential mosaic variants using NGS sequencing (49).

Despite the significant genomic similarities between ovarian and breast tumors and the existence of the HBOC disease entity, these tumors show some distinguishing features. Weber-Lassalle et al. subjected samples of HBOC patients to genetic analysis. The researchers identified germline variants in *BARD1* in breast cancer patients. Moreover, the presence of these variants was associated with an earlier onset of cancer (42.3 years) compared to the overall study group (48.6 years). *BARD1* variants were not detected in ovarian cancer (50).

## 5 Molecular diagnostics of patients diagnosed with ovarian cancer

It is extremely important that testing is not limited to *BRCA1/2*. The benefit of expanding molecular analyses was demonstrated by Tao et al. 25% of HGSOC patients had genetic alterations other than disorders leading to BRCA loss of function. This gives patients a chance to receive off-label treatment or other therapies described in this review as part of their participation in clinical trials (51).

Currently available tests to assess HRD include: analysis of genes associated with homologous recombination repair, genomic “scars” and/or mutational signatures, and real-time functional assessment of HRD. It should also be remembered that the HRD phenotype is very complex and thus extremely difficult to assess reliably and unequivocally in a single analysis. These validated tests can be used to make treatment decisions for PARPi. However, they are not effective in predicting the group of patients who will not benefit from PARPi, i.e. they have no negative predictive value (23, 52).

Considering the mutually exclusive nature of the occurrence of *BRCA* mutations and *CNNE1* amplification, the detection of high-level amplification in combination with *CCNE1* overexpression can be used as a criterion for predicting PARPi resistance (53). However, there are reports that genetic changes primarily thought to be mutually exclusive can co-occur. This is particularly true for loss of *RB* function, which appears to co-occur in tumors with *BRCA1/2* mutations or other disorders of homologous recombination pathway genes (54). Perhaps understanding this aspect will become possible through the use of spatial genomic, transcriptomic and proteomic technology. Spatial biology offers the possibility to analyze different cellular subclones and also complex interactions with the tumor microenvironment (TME), which together determine the process of carcinogenesis in an individual (55).

It should be noted that *CCNE1* overexpression can occur without copy number amplification. Clinical trials have demonstrated the efficacy of WEE1i in both patients with *CCNE1* amplification and overexpression. Therefore, future studies will probably evaluate the possibility of using *CCNE1* expression instead of amplification of this gene as a marker of susceptibility to WEE1i therapy (56).

It is also worth mentioning that the use of PARPi prolonged PFS, but not OS. Therefore, it is important to consider the use of another therapeutic option. Unfortunately, immunotherapies in ovarian cancer have been less successful than in non-small cell lung cancer and melanoma. In order to improve the efficacy of this therapy, patient selection based on immunological profiling including assessment of high-grade microsatellite instability (MSIH), mismatch repair deficiencies (dMMR) and HRD is proposed (57).

## 6 Molecular tests for HBOC

In order for a patient to qualify for genetic testing, a woman must meet criteria outlined in national guidelines. These include age, hormone receptor status in BC, OC histology, and history of cancers that may be related to HBOC in the family. Although, these guidelines in different countries cover the same aspects however, the cutoff points may vary. There is a need to refine consistent recommendations between countries for monitoring mutation carriers specifically in genes other than *BRCA1/2 (* 58).

The decision to use a multigene panel is up to the clinician. The physician, after analyzing the patient’s phenotype and taking a health history, can choose the most appropriate molecular diagnostic option to identify HBOC, or to exclude it with higher probability. In addition to commercially available panel tests with a wide range, some laboratories also offer custom-designed panels after listening to the clinician’s suggestions (45).

Despite improvements in diagnosis through the use of multigene panels, the issue of variants of uncertain significance (VUS) remains crucial. There is a need for algorithms as well as improved data flow and monitoring the situation globally to identify variants on which further research should be conducted to determine their clinical significance (59). These issues are discussed in more detail in the next section of the article.

Another important aspect is that HBOC tests should be tailored to different ethnic groups, as each has characteristic variants that occur with increased frequency compared to other groups. This approach can contribute to a better understanding of the molecular characteristics of specific communities of people and lead to the development of genetic tests dedicated to a specific community which will markedly improve the sensitivity and specificity of the test (60).

## 7 NGS – opportunity, challenges, analysis report and interpretation of results

With the advent of sequencing technologies, we have seen their continued evolution. Sanger sequencing, referred to as first-generation sequencing, is limited to regions of interest within the genes of interest. The development of next-generation sequencing technologies has enabled comprehensive sequencing of larger regions of the genome. In the case of gene panels, gene fragments whose genetic variation is responsible for the occurrence of a defined phenotype are sequenced. It is also possible to sequence targeted protein-coding regions of all genes - whole exome sequencing (WES) and even whole genome sequencing (WGS), i.e. coding and non-coding sequences. NGS allows for more extensive analyses and is consequently more efficient due to the time and cost savings compared to Sanger sequencing (61).

Using advances in technology such as NGS, it is possible to make treatment decisions (62). Currently, biopsy material archived in the form of formalin-fixed paraffin-embedded (FFPE) blocks is used for molecular diagnosis of solid tumors (63). In this material, due to the fixation process, there may be changes in nucleic acids, their degradation and also modification of nitrogenous bases, which will be misread as a sequence variant in NGS analysis. To prevent these errors, researchers are developing new and more efficient protocols for nucleic acid isolation and library preparation (64). Another aspect of crucial importance in the fight against ovarian cancer worldwide is early detection and also monitor disease progression based on molecular testing. Many studies are looking into the possibility of using liquid biopsy, which has the undoubted advantage of being non-invasive (62). Analysis of cell-free tumor DNA (ctDNA) circulating in the blood, which can be performed using the NGS technique, has many potential applications such as early detection of both primary disease and recurrence, predicting prognosis, characterization of tumor heterogeneity and monitoring of treatment over time (7, 65). However, the researchers point out that protocols for analyzing material obtained by liquid biopsy need to be refined, ctDNA enrichment methods need to be improved and the sensitivity of analytical techniques for detecting at low allele frequencies needs to be optimized. Further research is needed before liquid biopsy can be used in clinical practice (66).

The discovery of mutually exclusive pathways and also their interactions has become the basis for the development of new targeted therapies and, in the future, diagnostic regimens that qualify patients for treatment (67). Also in the case of approved drugs - PARPi, whose action is based on the concept of synthetic lethality, incoming data from studies show that it is essential to know the genetic context of the pathogenic change (68). For better insight and understanding of the clinical course of the disease, it is necessary to take into account not only individual genomic changes but their complex interrelationships, which together make up the characteristics of a given patient and thus determine the success of the planned therapeutic strategy (69). Consequently, there is a need to develop new biomarkers, the combined use of which will enable a better understanding of the patient’s clinical condition and allow the selection of the optimal treatment pathway (68).

NGS sequencing has provided a wealth of data regarding the molecular biology of cancers, including HGSOC. However, it is now known that the use of bioinformatics analysis and tools is essential. It is through these that the pathways of carcinogenesis can be analyzed in depth (67). To detect homologous recombination deficiency, research uses methods based on whole-exome or whole-genome sequencing. However, in the practice of molecular diagnostic oncology, targeted sequencing panels are most often used. Therefore, the number of identifiable mutations is too low for HRD signature analysis, and as a result, many cases with HRD are not identified and patients do not receive PARPi treatment. Gulhan et al. propose a computational tool called Signature Multivariate Analysis (SigMA), which enables detection of mutation signatures from panel sequencing data. The researchers’ impressive results indicate that the use of SigMA could significantly increase the number of patients who could be considered for targeted therapy (70). Schouten et al. developed a classifier that detects HRD-associated mutations based on evaluation of sequence variants in a panel of genes, epigenetic changes, and copy number alteration. The classifier proved to be an effective tool identifying most cancers with *BRCA* loss-of-function and *BRCA*-like phenotype, as well as all analyzed tumors with the *RAD51C* germline mutation (71). In contrast, Li et al. created a signature predictive of initial platinum resistance (IPR) not dependent on HRD but based on the differential expression of 11 genes. The researchers also identified significant differences in the characteristics of tumor infiltrating lymphocytes. The results suggest that patients with a high-IPR signature should show a better response to ICB (72).

In order for knowledge of the molecular profiles of tumors to be realistically reflected in clinical practice, that is, for treatment to be applied to a patient, it is important to keep in mind the significant heterogeneity of OC tumors, both temporally and spatially. For this reason, it seems reasonable that sequencing should also be performed after treatment and in relapse (73). This involves more research and thus cost generation. *In silico* data can be used to validate NGS assays instead of physical samples. In addition to high throughput and reliability, the use of *in silico* data makes it possible not to generate incremental costs associated with sequencing additional samples. These data can be successfully used to simulate hard-to-find gene variants, low-frequency variants or specific classes of variants (e.g., medium-sized insertions) (74).

The clinical course of the disease varies depending on the gene in which the pathogenic variant is present, whether the variant is germline, somatic, or an epigenetic modification, and the location of the change, i.e. which codon is affected (75). Therefore, correct and clear reporting and classify of detected changes is crucial. Somatic sequence variants are classified based on their clinical significance: tier I - variants of strong clinical significance; tier II - variants of potential clinical significance; tier III - variants of unknown clinical significance (VUS); and tier IV - variants of known non-significant significance (considered benign or probably benign). According to current guidelines, variants from tiers I-III must be included on the NGS analysis report, while it is not recommended to include lesions classified as tier IV. Tiers I and II have therapeutic, prognostic and diagnostic significance. VUS variants are the most challenging, and it is essential to report them which will enable researchers to decide later on their reclassification (76). Whereas germline variants are classified in a five-tier system: class 1 – non-pathogenic; class 2 - likely not pathogenic; class 3 – uncertain; class 4 - likely pathogenic; and class 5 - definitely pathogenic (77). Due to the distinctiveness of the scale used in the classification of somatic and germline sequential variants, it is helpful to include a verbal category name that indicates the pathogenicity of the variant to avoid potential confusion and misunderstanding.

Also extremely useful in the classification of variants are informatics tools that allow for the efficient flow of incoming data from NGS analyses. Hirotsu et al. demonstrated the utility of using MH BRCA and MH Guide developed by Molecular Health in determining the significance of VUS. The authors show significant value in confirming or exclusion of HBOC and also in predicting susceptibility to PARPi application when a variant of unknown clinical significance is detected (78).

## 8 Conclusions

**Figure 2:**
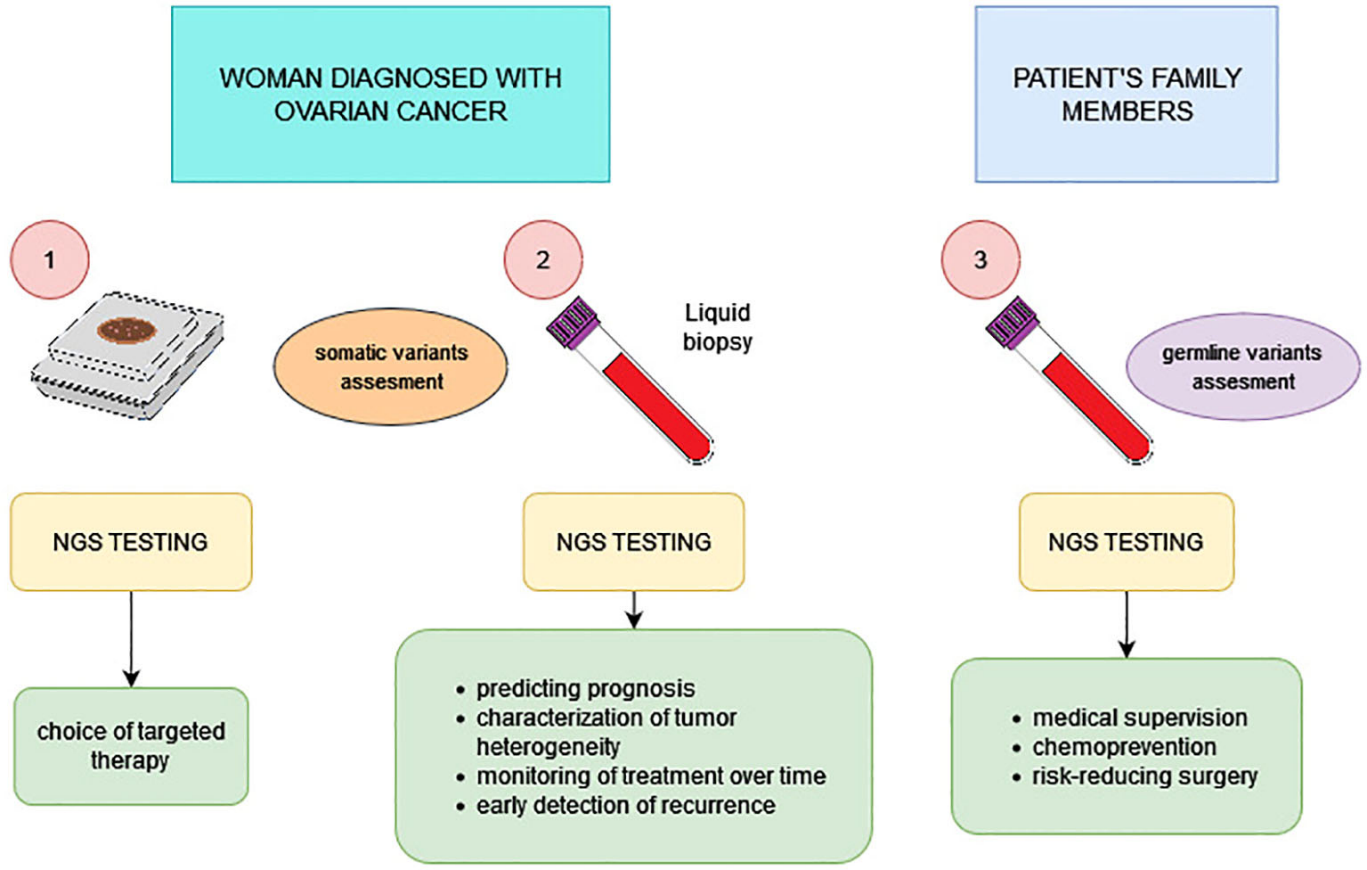
Possibilities of using NGS technology in the diagnostic and therapeutic process of ovarian cancer.

Modern medicine has a great knowledge of the molecular-level changes involved in carcinogenesis, resulting in breakthroughs in patient diagnosis and treatment. Approaching cancer as if it were a disease determined by genetic changes and using NGS technology can significantly improve the prognosis of women with HGSOC. The use and utility of NGS testing in OC is summarized in **Figure 2**. However, despite its high efficiency, performance, NGS poses many challenges. Bioinformatics tools and the use of algorithms to obtain comprehensive and reliable molecular characterization of a patient’s sample are helpful. It is crucial to properly interpret the test results and relate them to the patient’s clinical condition. There is also a need to search for new therapeutic targets and conduct clinical trials of further targeted therapies. Before introducing new drugs into clinical practice, it is necessary to evaluate their efficacy, selectivity against tumor cells and also possible side effects. Clinical trials on the efficacy of new molecularly targeted drugs are being conducted around the world and **Table 2** collects examples of such trials. A great opportunity may be the possibility of tumor-agnostic therapy, i.e. the use of targeted drugs regardless of the type of cancer just on the basis of finding the presence of a specific genetic change. Biomarkers should be sought to predict the response to the planned therapy. A very important aspect is the identification of women with a hereditary predisposition to ovarian cancer. As the range of therapeutic options grows, it is necessary to develop a laboratory diagnostic pathway that allows the best classification of patients for specific therapies. Pursuing these goals will contribute to more efficient diagnosis and treatment from an economic perspective and, invaluably, to improving the quality and prolonging the lives of patients. This can only be achieved through continuous improvement of skills and cooperation between clinicians and laboratory diagnosticians.

**Table 2:** Clinical trials of drugs that can be used to treat ovarian cancer.

NCT number	Official study title	Study phase
PLK1 inhibitor (Polo-like Kinase 1 inhibitor)		
NCT01121406	Phase II Randomized Trial of the Polo-like Kinase 1 Inhibitor BI 6727 Monotherapy Versus Investigator´s Choice Chemotherapy in Ovarian Cancer Patients Resistant or Refractory to Platinum-based Cytotoxic Therapy	Phase 2
PI3K inhibitor (phosphoinositide 3-kinase inhibitor)		
NCT05768139	First-in-Human Study of STX-478, a Mutant-Selective PI3Kα Inhibitor as Monotherapy and in Combination With Other Antineoplastic Agents in Participants With Advanced Solid Tumor	Phase 1 Phase 2
NCT05295589	A Randomized Phase II Trial Comparing the Combination of PI3K Inhibitor Copanlisib (BAY 80-6946) and PARP Inhibitor Olaparib (AZD2281) to Standard Chemotherapy in Patients With Recurrent Platinum Resistant Ovarian, Fallopian Tube, or Primary Peritoneal Cancer Who Have Progressed Through Prior PARP Inhibitor Therapy	Phase 2
NCT02476955	An Open-label Phase 1b Study of ARQ 092 in Combination With Other Antineoplastic Agents in Subjects With Selected Solid Tumors	Phase 1
NCT01363232	A Phase Ib, Open-label, Multi-center, Dose-escalation and Expansion Study of an Orally Administered Combination of BKM120 Plus MEK162 in Adult Patients With Selected Advanced Solid Tumors	Phase 1
NCT02307240	Phase I Open Label, Multi-center Study to Assess the Safety, Tolerability and Pharmacokinetics of Orally Administered CUDC-907, an HDAC and PI3K Inhibitor, in Subjects With Advanced/Relapsed Solid Tumors	Phase 1
NCT03586661	A Phase Ib Study of the Oral PARP Inhibitor Niraparib With the Intravenous PI3K Inhibitor Copanlisib for Recurrent Endometrial and Recurrent Ovarian, Primary Peritoneal, or Fallopian Tube Cancer	Phase 1
NCT05082025	Phase 2 Study of PI3K Inhibitor Copanlisib in Combination With Fulvestrant in Selected ER+ and/or PR+ Cancers With PI3K (PIK3CA, PIK3R1) and/or PTEN Alterations	Phase 2
NCT04586335	Open Label, Phase Ib Study to Evaluate the Safety, Tolerability, Pharmacokinetics and Clinical Activity of CYH33, an Oral PI3K Inhibitor in Combination With Olaparib, an Oral PARP Inhibitor in Patients With Advanced Solid Tumors	Phase 1
NCT05683418	A Study to Evaluate the Safety and Tolerability of the Covalent Phosphoinositide-3-Kinase (PI3K)-Alpha Inhibitor, TOS-358, in Adult Subjects With Select Solid Tumors	Phase 1
NCT01363232	A Phase Ib, Open-label, Multi-center, Dose-escalation and Expansion Study of an Orally Administered Combination of BKM120 Plus MEK162 in Adult Patients With Selected Advanced Solid Tumors	Phase 1
AKT inhibitor (protein kinase B inhibitor)		
NCT01226316	A Phase I, Open-Label, Multicentre Study to Assess the Safety, Tolerability, Pharmacokinetics and Preliminary Anti-tumour Activity of Ascending Doses of AZD5363 Under Adaptable Dosing Schedules in Patients With Advanced Solid Malignancies	Phase 1
NCT04055649	Phase II Study of ONC201 Plus Weekly Paclitaxel in Patients With Platinum-Resistant Refractory or Recurrent Epithelial Ovarian, Fallopian Tube, or Primary Peritoneal Cancer	Phase 2
NCT01283035	A Phase II Study of MK-2206 in the Treatment of Recurrent High-Grade Serous Platinum-Resistant Ovarian, Fallopian Tube, or Primary Peritoneal Cancer	Phase 2
NCT00431054	Pharmacodynamic Trial: Molecular Marker & Imaging Studies as Primary Endpoints to Determine Optimal Biological Dosage of Perifosine, Orally Avail AKT PH Domain Inhibitor Combined w/ Docetaxel in Patients w/Relapsed Epithelial Ovarian Cancer	Phase 1
NCT01653912	An Open-Label Phase I/II Study of GSK2110183 in Combination With Carboplatin and Paclitaxel in Subjects With Platinum-Resistant Ovarian Cancer	Phase 1 Phase 2
NCT01690468	A Phase IA/IB Trial of PTX-200 and Carboplatin in Patients With Platinum-Resistant Recurrent Ovarian Cancer	Phase 1 Phase 2
NCT02476955	An Open-label Phase 1b Study of ARQ 092 in Combination With Other Antineoplastic Agents in Subjects With Selected Solid Tumors	Phase 1
NCT01266954	An Open Label Study To Investigate the Pharmacokinetics and Pharmacodynamics of Repeat Escalating Doses of the Oral AKT Inhibitor GSK2141795 by 18F FDG PET Analysis in Subjects With Ovarian Cancer	Phase 1
EGFR inhibitor (epidermal growth factor receptor inhibitor)		
NCT00049556	Phase II Pilot Study of Clinical Activity and Proteomic Pathway Profiling of the EGFR Inhibitor, ZD1839 (Iressa; Gefitinib), in Patients With Epithelial Ovarian Cancer or Cervical Cancer	Phase 2
BET inhibitor (bromodomain and extraterminal domain inhibitor)		
NCT02711137	A Phase 1/2, Open-Label, Dose-Escalation/Dose-Expansion, Safety and Tolerability Study of INCB057643 in Subjects With Advanced Malignancies	Phase 1 Phase 2
NCT05252390	Phase 1/2 Safety and Efficacy Study of NUV-868 as Monotherapy and in Combination With Olaparib or Enzalutamide in Adult Patients With Advanced Solid Tumors	Phase 1 Phase 2
NCT04840589	Phase I/Ib Trial Evaluating the Safety and Efficacy of BET Inhibitor, ZEN003694 With PD-1 Inhibitor, Nivolumab With or Without CTLA-4 Inhibitor, Ipilimumab in Solid Tumors	Phase 1
NCT05071937	Phase ll Study of a BET Inhibitor, ZEN003694, Combined With a PARP Inhibitor, Talazoparib, in Patients With Recurrent Ovarian Cancer	Phase 2
NCT05950464	A Phase 1B Study of Combination ATR (M1774) and BET Inhibition (ZEN00-3694) to Exploit ARID1A Loss in Recurrent Ovarian and Endometrial Cancer	Phase 1
DNMT inhibitor (DNA methyltransferase inhibitor)		
NCT03206047	A Randomized Phase 2 Trial of Atezolizumab (MPDL3280A), SGI-110 and CDX-1401 Vaccine in Recurrent Ovarian Cancer	Phase 1 Phase 2
NCT02901899	An Open Label Phase II Trial of Guadecitabine and Pembrolizumab in Platinum Resistant Recurrent Ovarian Cancer	Phase 2
HDAC inhibitor (histone deacetylase inhibitor)		
NCT00045006	Phase I Clinical Trial of Oral Suberoylanilide Hydroxamic Acid - SAHA (MSK390) in Patients With Advanced Solid Tumors and Hematologic Malignancies	Phase 1
NCT00413075	Open Label, Dose Escalation Trial of Oral PXD101 in Patients With Advanced Solid Tumors	Phase 1
NCT00413322	A Phase I Safety, Pharmacodynamic, Anti-Tumor Activity, and Pharmacokinetic Study of PXD101 Alone and in Combination With 5-Fluorouracil in Patients With Advanced Solid Tumors	Phase 1
NCT00993616	A Phase II Evaluation of Belinostat (NSC #726630) and Carboplatin (NSC #241240) in the Treatment of Recurrent or Persistent Platinum-Resistant Ovarian, Fallopian Tube, or Primary Peritoneal Cancer	Phase 2
NCT00020579	A Phase I Study of an Oral Histone Deacetylase Inhibitor, MS-275, in Refractory Solid Tumors and Lymphomas	Phase 1
NCT00421889	A Phase I/II Safety, Pharmacodynamic, and Pharmacokinetic Study of Intravenously Administered PXD101 Plus Carboplatin or Paclitaxel or Both in Patients With Advanced Solid Tumours	Phase 1 Phase 2
NCT04703920	A Phase 1 Dose-Escalation Trial of Talazoparib in Combination With Belinostat for Metastatic Breast Cancer, Metastatic Castration Resistant Prostate Cancer, and Metastatic Ovarian Cancer	Phase 1
NCT00132067	A Phase II Evaluation of Vorinostat, (SAHA, NCI-Supplied Agent [NSC #701852]) in the Treatment of Persistent or Recurrent Epithelial Ovarian or Primary Peritoneal Carcinoma	Phase 2
NCT02915523	A Randomized, Placebo-controlled, Double-blind, Multicenter Phase 1b/2 Study of Avelumab With or Without Entinostat in Patients With Advanced Epithelial Ovarian Cancer Which Has Progressed or Recurred After First-line Platinum-based Chemotherapy and at Least Two Subsequent Lines of Treatment With a Safety Lead-in	Phase 1 Phase 2
NCT00301756	A Phase 2 Study of PXD101 in Platinum Resistant Epithelial Ovarian Tumors and Micropapillary/Borderline (LMP) Ovarian Tumors	Phase 2
NCT02728492	Open-label Multicenter Multiple Ascending Dose Study to Evaluate Safety, Tolerability and Pharmacokinetics of Quisinostat, a Histone Deacetylase Inhibitor, in Combination With Gemcitabine + Cisplatin Chemotherapy (Second Line for Patients With Non-small Cell Lung Cancer) or Paclitaxel + Carboplatin Chemotherapy (Second Line for Patients With Non-small-cell Lung Cancer, Second and Subsequent Lines for Patients With Epithelial Ovarian Cancer)	Phase 1
NCT02948075	Multicenter, Open-label Study of Safety and Efficacy of Quisinostat in Combination With Paclitaxel + Carboplatin Chemotherapy in Patients With Metastatic or Locally Advanced Epithelial Ovarian Cancer, Primarily Peritoneal or Fallopian Tube Carcinoma, Resistant to First Line Platinum and Paclitaxel Based Chemotherapy	Phase 2
NCT02307240	Phase I Open Label, Multi-center Study to Assess the Safety, Tolerability and Pharmacokinetics of Orally Administered CUDC-907, an HDAC and PI3K Inhibitor, in Subjects With Advanced/Relapsed Solid Tumors	Phase 1
NCT04315233	A Phase I/Ib Trial of the CDK4/6 Antagonist Ribociclib And The HDAC Inhibitor Belinostat In Patients With Metastatic Triple Negative Breast Cancer And Recurrent Ovarian Cancer With Response Prediction By Genomics (CHARGE)	Phase 1
PARG inhibitor (poly(ADP-ribose) glycohydrolase inhibitor)		
NCT05787587	A Study of PARG Inhibitor IDE161 in Participants With Advanced Solid Tumors	Phase 1
CDK2 inhibitor (cyclin-dependent kinase 2 inhibitor)		
NCT04553133	Phase 1/2A Dose Escalation, Finding And Expansion Study Evaluating Safety, Tolerability, Pharmacokinetics, Pharmacodynamics And Anti Tumor Activity Of PF-07104091 As A Single Agent And In Combination Therapy	Phase 1 Phase 2
NCT05905341	A Phase 1, Open-Label, Multicenter, Dose Escalation And Dose Expansion Study To Evaluate The Safety, Tolerability, Pharmacokinetics, Pharmacodynamics, And Antitumor Activity of PF-07224826, As A Single Agent And In Combination With Endocrine Therapy In Participants With Advanced Solid Tumors	Phase 1
PKMYT1 inhibitor (Protein Kinase, Membrane Associated Tyrosine/Threonine 1 inhibitior)		
NCT04855656	Phase 1 Study of the Safety, Pharmacokinetics, Pharmacodynamics and Preliminary Clinical Activity of RP-6306 Alone or in Combination With RP-3500 in Patients With Advanced Solid Tumors	Phase 1
NCT05147272	Phase 1 Study of the PKMYT1 Inhibitor RP-6306 in Combination With Gemcitabine for the Treatment of Advanced Solid Tumors (MAGNETIC Study)	Phase 1
NCT05147350	Phase 1 Study of the PKMYT1 Inhibitor RP-6306 in Combination With FOLFIRI for the Treatment of Advanced Solid Tumors	Phase 1
WEE1 inhibitor		
NCT03345784	A Phase I Study of the Wee 1 Kinase (Wee 1) Inhibitor AZD1775 in Combination With Radiotherapy and Cisplatin in Cervical, Upper Vaginal and Uterine Cancers (10041848, 10008224, 10008238, 10046888, 10014735)	Phase 1
NCT01164995	Phase II Pharmacological Study With Wee-1 Inhibitor MK-1775 Combined With Carboplatin in Patients With p53 Mutated Epithelial Ovarian Cancer and Early Relapse (< 3 Months) or Progression During Standard First Line Treatment	Phase 2
NCT04768868	A Phase 1, Open-Label, Multi-Center, Dose Escalation and Expansion Study to Evaluate Safety, Tolerability, Pharmacokinetics, and Anti-Tumor Activity of the WEE1 Inhibitor IMP7068 Monotherapy in Patients With Advanced Solid Tumors	Phase 1
NCT05368506	An Early Phase I Study of the Pharmacodynamics of WEE1 Inhibitor, ZN-c3, in Metastatic Solid Tumors	Early Phase 1
NCT02101775	A Randomized Placebo-Controlled Phase II Trial Comparing Gemcitabine Monotherapy to Gemcitabine in Combination With AZD 1775 (MK 1775) in Women With Recurrent, Platinum Resistant Epithelial Ovarian, Primary Peritoneal, or Fallopian Tube Cancers	Phase 2
ATR inhibitor		
NCT04657068	A Phase I/IIa, Open-label, Multi-center Study to Assess the Safety, Tolerability, Pharmacokinetics and Preliminary Efficacy of the ATR Kinase Inhibitor ART0380 Administered Orally as Monotherapy and in Combination to Patients With Advanced or Metastatic Solid Tumors	Phase 1 Phase 2
NCT04491942	A Phase 1 Trial of the ATR Inhibitor BAY 1895344 in Combination With Cisplatin and With Cisplatin Plus Gemcitabine in Advanced Solid Tumors With an Emphasis on Urothelial Carcinoma	Phase 1
NCT05950464	A Phase 1B Study of Combination ATR (M1774) and BET Inhibition (ZEN00-3694) to Exploit ARID1A Loss in Recurrent Ovarian and Endometrial Cancer	Phase 1
NCT04149145	Trial of M4344 and Niraparib in Patients With PARP Resistant Recurrent Ovarian Cancer	Phase 1
NCT03462342	Combination ATR and PARP Inhibitor (CAPRI) Trial With AZD 6738 and Olaparib in Recurrent Ovarian Cancer	Phase 2
NCT02487095	A Phase I/II Trial of Topotecan With VX-970 (M6620), an ATR Kinase Inhibitor in Small Cell Cancers	Phase 1 Phase 2
NCT04267939	An Open-label Phase 1b Study to Determine the Maximum Tolerated and/or Recommended Phase 2 Dose of the ATR Inhibitor Elimusertib (BAY 1895344) in Combination With PARP Inhibitor Niraparib, in Participants With Recurrent Advanced Solid Tumors and Ovarian Cancer	Phase 1
NCT04616534	Phase 1 Trial of Gemcitabine Combined With the Elimusertib (BAY 1895344) ATR Inhibitor With Expansion Cohorts in Advanced Pancreatic and Ovarian Cancer	Phase 1
NCT04065269	ATARI: ATr Inhibitor in Combination With Olaparib in Gynaecological Cancers With ARId1A Loss	Phase 2
NCT02595892	Phase 2 Study of M6620 (VX-970) in Combination With Gemcitabine Versus Gemcitabine Alone in Subjects With Platinum-Resistant Recurrent Ovarian or Primary Peritoneal Fallopian Tube Cancer	Phase 2
Aurora kinase inhibitor		
NCT01091428	Randomized Phase 2 Study of MLN8237, an Aurora A Kinase Inhibitor, Plus Weekly Paclitaxel or Weekly Paclitaxel Alone in Patients With Recurrent Epithelial Ovarian, Fallopian Tube, or Primary Peritoneal Cancer, Preceded by a Phase 1 Portion in Patients With Ovarian or Breast Cancer	Phase 2
NCT00853307	A Phase 2 Study of MLN8237, a Novel Aurora A Kinase Inhibitor, in the Treatment of Patients With Platinum-Refractory or Platinum-Resistant Epithelial Ovarian, Fallopian Tube, or Primary Peritoneal Carcinoma	Phase 2
mTORC1/2 inhibitor		
NCT01149434	Drug- Drug Interaction Study of JI-101 & Everolimus in Advanced Solid Tumors, Expansion Pharmacodynamic Study of JI-101 in Advanced Low Grade Endocrine Tumors, Ovarian Cancers or K-RAS Mutant Colon Cancers	Phase 1 Phase 2
NCT02208375	A Phase Ib Study of the Oral PARP Inhibitor Olaparib With the Oral mTORC1/2 Inhibitor AZD2014 or the Oral AKT Inhibitor AZD5363 for Recurrent Endometrial, Triple Negative Breast, and Ovarian, Primary Peritoneal, or Fallopian Tube Cancer	Phase 1 Phase 2
NCT04998760	A Multi-center Clinical Study to Evaluate Dual mTORC1/2 Inhibitor (ATG 008) or Selective Inhibitor of Nuclear Export Compound (ATG-010) in Combination With Chemotherapy in Patients With Relapsed or Metastatic Ovarian Cancer, Endometrial Cancer, and Cervical Cancer	Phase 2
CDK7 inhibitor (cyclin-dependent kinase 7 inhibitor)		
NCT04726332	A Dose Escalation and Expansion Study of the Safety and Pharmacokinetics of XL102 as Single-Agent and Combination Therapy in Subjects With Inoperable Locally Advanced or Metastatic Solid Tumors	Phase 1
NCT03134638	A Phase 1 Study of SY-1365, a Selective CDK7 Inhibitor, in Adult Patients With Advanced Solid Tumors	Phase 1

Data from ClinicalTrials.gov available as of July 31, 2023.

## Author contributions

PB: Conceptualization, Data collection, Writing. JK: Supervision. JN: Supervision. All authors contributed to the article and approved the submitted version.

## References

[1] Khanlarkhani N Azizi E Amidi F Khodarahmian M Salehi E Pazhohan A. Metabolic risk factors of ovarian cancer: a review. JBRA Assisted Reprod (2022) 26(2):335–47. doi: 10.5935/1518-0557.20210067 PMC9118962 34751020

[2] Huang J Chan WC Ngai CH Lok V Zhang L Lucero-Prisno DE III. Worldwide burden, risk factors, and temporal trends of ovarian cancer: A global study. Cancers (2022) 14:2230. doi: 10.3390/cancers14092230 35565359 PMC9102475

[3] Otsuka I. Mechanisms of high-grade serous carcinogenesis in the fallopian tube and ovary: current hypotheses, etiologic factors, and molecular alterations. Int J Mol Sci (2021) 22:4409. doi: 10.3390/ijms22094409 33922503 PMC8122889

[4] Bellcross CA. Hereditary breast and ovarian cancer. An updated primer for OB/GYNs. Obstet Gynecol Clin N Am (2022) 49:117–47. doi: 10.1016/j.ogc.2021.11.005 35168766

[5] Serio PAdMP de Lima Pereira GF Katayama MLH Roela RA Maistro S Folgueira MAAK. Somatic mutational profile of high-grade serous ovarian carcinoma and triple-negative breast carcinoma in young and elderly patients: similarities and divergences. Cells (2021) 10:3585. doi: 10.3390/cells10123586 34944094 PMC8700427

[6] Yoshida R. Hereditary breast and ovarian cancer (HBOC): review of its molecular characteristics, screening, treatment, and prognosis. Breast Cancer (2021) 28:1167–80. doi: 10.1007/s12282-020-01148-2 PMC8514387 32862296

[7] Morganti S Tarantino P Ferraro E D’Amico P Viale G Trapani D. Complexity of genome sequencing and reporting: Next generation sequencing (NGS) technologies and implementation of precision medicine in real life. Crit Rev Oncol/Hematol (2019) 133:171–82. doi: 10.1016/j.critrevonc.2018.11.008 30661654

[8] Hussen BM Abdullah ST Salihi A Sabir DK Sidiq KR Rasul MF. The emerging roles of NGS in clinical oncology and personalized medicine. Pathol - Res Pract (2022) 230:153760. doi: 10.1016/j.prp.2022.153760 35033746

[9] Zhong Y Xu F Wu J Schubert J Li MM. Application of next generation sequencing in laboratory medicine. Ann Lab Med (2021) 41:25–43. doi: 10.3343/alm.2021.41.1.25 32829577 PMC7443516

[10] Lheureux S Gourley C Vergote I Oza AM. Epithelial ovarian cancer. Lancet (2019) 393:1240–53. doi: 10.1016/S0140-6736(18)32552-2 30910306

[11] Handley KF Sims TT Bateman NW Glassman D Foster KI Lee S. Classification of high-grade serous ovarian cancer using tumor morphologic characteristics. JAMA Network Open (2022) 5:e2236626. doi: 10.1001/jamanetworkopen.2022.36626 36239936 PMC9568802

[12] Khashaba M Fawzy M Abdel-Aziz A Eladawei G Nagib R. Subtyping of high grade serous ovarian carcinoma: histopathological and immunohistochemical approach. J Egyptian Natl Cancer Inst (2022) 34(6). doi: 10.1186/s43046-022-00104-9 PMC8827304 35138498

[13] Lheureux S Braunstein M Oza AM. Epithelial ovarian cancer: evolution of management in the era of precision medicine. CA Cancer J Clin (2019) 69:280–304. doi: 10.3322/caac.21559 31099893

[14] Harbin LM Gallion HH Allison DB Kolesar JM. Next generation sequencing and molecular biomarkers in ovarian cancer—An opportunity for targeted therapy. Diagnostics (2022) 12:842. doi: 10.3390/diagnostics12040842 35453890 PMC9030726

[15] Xu H George E Kinose Y Kim H Shah JB Peake JD. CCNE1 copy number is a biomarker for response to combination WEE1-ATR inhibition in ovarian and endometrial cancer models. Cell Rep Med (2021) 2:100394. doi: 10.1016/j.xcrm.2021.100394 34622231 PMC8484689

[16] Gorski JW Ueland FR Kolesar JM. CCNE1 amplification as a predictive biomarker of chemotherapy resistance in epithelial ovarian cancer. Diagnostics (2020) 10:279. doi: 10.3390/diagnostics10050279 32380689 PMC7277958

[17] da Costa AABA do Canto LM Larsen SJ Ribeiro ARG Stecca CE Petersen AH. Genomic profiling in ovarian cancer retreated with platinum based chemotherapy presented homologous recombination deficiency and copy number imbalances of CCNE1 and RB1 genes. BMC Cancer (2019) 19:422. doi: 10.1186/s12885-019-5622-4 31060523 PMC6503431

[18] Pulliam N Fang F Ozes AR Tang J Adewuyi A Keer H. An effective epigenetic-PARP inhibitor combination therapy for breast and ovarian cancers independent of BRCA-mutations. Clin Cancer Res (2018) 24:3163–75. doi: 10.1158/1078-0432.CCR-18-0204 PMC7003715 29615458

[19] Gasimli K Raab M Rad MT Kurunci-Csacsko E Becker S Strebhardt K. Sequential targeting of PLK1 and PARP1 reverses the resistance to PARP inhibitors and enhances platin-based chemotherapy in BRCA-deficient high-grade serous ovarian cancer with KRAS amplification int. J Mol Sci (2022) 23:10892. doi: 10.3390/ijms231810892 PMC9502276 36142803

[20] Staropoli N Ciliberto D Giudice TD Iuliano E Cucè M Grillone F. The Era of PARP inhibitors in ovarian cancer: “Class Action” or not? A systematic review and meta-analysis. Crit Rev Oncol/Hematol (2018) 131:83–9. doi: 10.1016/j.critrevonc.2018.08.011 30293710

[21] Slade D. PARP and PARG inhibitors in cancer treatment. Genes Dev (2020) 34:360–94. doi: 10.1101/gad.334516.119 PMC7050487 32029455

[22] Cong K Peng M Kousholt AN Lee WTC Lee S Nayak S. Replication gaps are a key determinant of PARP inhibitor synthetic lethality with BRCA deficiency. Mol Cell (2021) 81:3128–3144.e7. doi: 10.1016/j.molcel.2021.06.011 34216544 PMC9089372

[23] Miller RE Elyashiv O El-Shakankery KH Ledermann JA. Ovarian cancer therapy: homologous recombination deficiency as a predictive biomarker of response to PARP inhibitors. OncoTargets Ther (2022) 15:1105–17. doi: 10.2147/OTT.S272199 PMC9547601 36217436

[24] Miller RE. Poly(ADP-ribose) polymerase inhibitor combination therapy. Cancer J (2021) 27(6):506–10. doi: 10.1097/PPO.0000000000000565 34904814

[25] Secord AA O’Malley DM Sood AK Westin SN Liu JF. Rationale for combination PARP inhibitor and antiangiogenic treatment in advanced epithelial ovarian cancer: A review. Gynecol Oncol (2021) 162:482–95. doi: 10.1016/j.ygyno.2021.05.018 34090705

[26] Kraya AA Maxwell KN Eiva MA Wubbenhorst B Pluta J Feldman M. PTEN loss and BRCA1 promoter hypermethylation negatively predict for immunogenicity in BRCA-deficient ovarian cancer. JCO Precis Oncol (2022) 6:e2100159. doi: 10.1200/PO.21.00159 35201851 PMC8982238

[27] Shen S Wang G Zhang R Zhao Y Yu H Wei Y. Development and validation of an immune gene-set based Prognostic signature in ovarian cancer. EBioMedicine (2019) 40:318–26. doi: 10.1016/j.ebiom.2018.12.054 PMC6412087 30594555

[28] Gupta VG Hirst J Petersen S Roby KF Kusch M Zhou H. Entinostat, a selective HDAC1/2 inhibitor, potentiates the effects of olaparib in homologous recombination proficient ovarian cancer. Gynecol Oncol (2021) 162:163–72. doi: 10.1016/j.ygyno.2021.04.015 PMC8647995 33867143

[29] Pillay N Brady RM Dey M Morgan RD Taylor SS. DNA replication stress and emerging prospects for PARG inhibitors in ovarian cancer therapy. Prog Biophys Mol Biol (2021) 163:160–70. doi: 10.1016/j.pbiomolbio.2021.01.004 33524442

[30] van Wagensveld L van Baal JOAM Timmermans M Gaillard D Borghuis L Coffelt SB. Homologous recombination deficiency and cyclin E1 amplification are correlated with immune cell infiltration and survival in high-grade serous ovarian cancer. Cancers (2022) 14:5965. doi: 10.3390/cancers14235965 36497449 PMC9738162

[31] ShuyangYao F Meric-Bernstam Hong D Janku F Naing A Piha-Paul S. Clinical characteristics and outcomes of phase I cancer patients with CCNE1 amplifcation: MD Anderson experiences. Scic Rep (2022) 12:8701. doi: 10.1038/s41598-022-12669-5 PMC9130298 35610322

[32] Tadesse S Anshabo AT Portman N Lim E Tilley W Caldon CE. Targeting CDK2 in cancer: challenges and opportunities for therapy. Drug Discov Today (2020) 25:406–13. doi: 10.1016/j.drudis.2019.12.001 31839441

[33] Gallo D Young JTF Fourtounis J Martino G Álvarez-Quilón A Bernier C. CCNE1 amplification is synthetic lethal with PKMYT1 kinase inhibition. Nature (2022) 604:749–56. doi: 10.1038/s41586-022-04638-9 PMC9046089 35444283

[34] Goehring L Huang TT. WEE1i-ATRi combination therapy: a promising low-dose treatment for CCNE1-amplified gynecologic cancers. Cell Rep Med (2021) 2:100402. doi: 10.1016/j.xcrm.2021.100402 34622238 PMC8484682

[35] Li S Wang T Fei X Zhang M. ATR inhibitors in platinum-resistant ovarian cancer. Cancers (2022) 14:5902. doi: 10.3390/cancers14235902 36497387 PMC9740197

[36] Du X Li J Luo X Li R Li F Zhang Y. Structure-activity relationships of Wee1 inhibitors: A review. Eur J Med Chem (2020) 203:112524. doi: 10.1016/j.ejmech.2020.112524 32688199

[37] Martins FC Couturier D-L de Santiago I Sauer CM Vias M Angelova M. Clonal somatic copy number altered driver events inform drug sensitivity in high-grade serous ovarian cancer. Nat Commun (2022) 13:6360. doi: 10.1038/s41467-022-33870-0 36289203 PMC9606297

[38] Martins FC Couturier D-L d. Santiago I Vias M Sanders D Piskorz A. Combination of mTOR inhibition and paclitaxel as a personalised strategy in the context of MYC-amplified high-grade serous ovarian cancer. Ann Oncol (2019) 30:vii10–1. doi: 10.1093/annonc/mdz413.038

[39] Zeng M Kwiatkowski NP Zhang T Nabet B Xu M Liang Y. Targeting MYC dependency in ovarian cancer through inhibition of CDK7 and CDK12/13. eLife (2018) 7:e39030. doi: 10.7554/eLife.39030 30422115 PMC6251623

[40] Gong X Du J Parsons SH Merzoug FF Webster Y Iversen PW. Aurora A kinase inhibition is synthetic lethal with loss of the RB1 tumor suppressor gene. Cancer Discov (2019) 9:248–63. doi: 10.1158/2159-8290.CD-18-0469 30373917

[41] Oser MG Fonseca R Chakraborty AA Brough R Spektor A Jennings RB. Cells lacking the RB1 tumor suppressor gene are hyperdependent on aurora B kinase for survival. Cancer Discov (2019) 9(2):230–47. doi: 10.1158/2159-8290.CD-18-0389 PMC6368871 30373918

[42] Samuel D Diaz-Barbe A Pinto A Schlumbrecht M George S. Hereditary ovarian carcinoma: cancer pathogenesis looking beyond BRCA1 and BRCA2. Cells (2022) 11(539). doi: 10.3390/cells11030539 PMC8834207 35159349

[43] Schettini F Giudici F Bernocchi O Sirico M Corona SP Giuliano M. Poly (ADP-ribose) polymerase inhibitors in solid tumours: Systematic review and meta-analysis. Eur J Cancer (2021) 149:134–52. doi: 10.1016/j.ejca.2021.02.035 33862496

[44] Yamauchi H Takei J. Management of hereditary breast and ovarian cancer. Int J Clin Oncol (2018) 23:45–51. doi: 10.1007/s10147-017-1208-9 29185095

[45] Petrucelli N Daly MB Pal T. BRCA1- and BRCA2-associated hereditary breast and ovarian cancer. GeneReviews (1998). 20301425

[46] Doddato G Valentino F Giliberti A Papa FT Tita R Bruno LP. Exome sequencing in BRCA1-2 candidate familias: the contribution of other cancer susceptibility genes. Front Oncol (2021) 11:649435. doi: 10.3389/fonc.2021.649435 34026625 PMC8139251

[47] Velázquez C Lastra E Cobos FA Abella L de la Cruz V Hernando BA. A comprehensive custom panel evaluation for routine hereditary cancer testing: improving the yield of germline mutation detection. J Transl Med (2020) 18(232). doi: 10.1186/s12967-020-02391-z PMC7288470 32522261

[48] Resch LD Hotz A Zimmer AD Komlosi K Singh N Tzschach A. The importance of extended analysis using current molecular genetic methods based on the example of a cohort of 228 patients with hereditary breast and ovarian cancer syndrome. Genes (2021) 12:1483. doi: 10.3390/genes12101483 34680878 PMC8535571

[49] Mayoral IH Santiago AA Sánchez-Zapardiel JM Calero BH de la Hoya M Gómez-Sanz A. Unexpected findings in hereditary breast and ovarian cancer syndrome: low-level constitutional mosaicism in BRCA2. Genes (2023) 14:502. doi: 10.3390/genes14020502 36833429 PMC9957471

[50] Weber-Lassalle N Borde J Weber-Lassalle K Horváth J Niederacher D Arnold N. Germline loss-of-function variants in the BARD1 gene are associated with early-onset familial breast cancer but not ovarian cancer. Breast Cancer Res (2019) 21:55. doi: 10.1186/s13058-019-1137-9 31036035 PMC6489184

[51] Tao J Sun D Zhou H Zhu J Zhang X Hou H. Next-generation sequencing identifies potential novel therapeutic targets in Chinese HGSOC patients. Pathol - Res Pract (2022) 238:154074. doi: 10.1016/j.prp.2022.154074 35988354

[52] Miller RE Leary A Scott CL Serra V Lord CJ Bowtell D. ESMO recommendations on predictive biomarker testing for homologous recombination deficiency and PARP inhibitor benefit in ovarian cancer. Ann Oncol (2020) 31(12):1606–22. doi: 10.1016/j.annonc.2020.08.2102 33004253

[53] Chan AM Enwere E McIntyre JB Wilson H Nwaroh C Wiebe N. Combined CCNE1 high-level amplification and overexpression is associated with unfavourable outcome in tubo-ovarian high-grade serous carcinoma. J Pathol Clin Res (2020) 6:252–62. doi: 10.1002/cjp2.168 PMC7578325 32391646

[54] Hollis RL. Molecular characteristics and clinical behaviour of epithelial ovarian cancers. Cancer Lett (2023) 555:216057. doi: 10.1016/j.canlet.2023.216057 36627048

[55] Seferbekova Z Lomakin A Yates LR Gerstung M. Spatial biology of cancer evolution. Nat Rev Genet (2023) 24:295–313. doi: 10.1038/s41576-022-00553-x 36494509

[56] Takebe N Naqash AR Coyne GOS Kummar S Do K Bruns A. Safety, anti-tumor activity, and biomarker analysis in a phase 1 trial of the once-daily Wee1 inhibitor adavosertib (AZD1775) in patients with advanced solid tumors. Clin Cancer Res (2021) 27:3834–44. doi: 10.1158/1078-0432.CCR-21-0329 PMC8282703 33863809

[57] Morand S Devanaboyina M Staats H Stanbery L Nemunaitis J. Ovarian cancer immunotherapy and personalized medicine. Int J Mol Sci (2021) 22:6532. doi: 10.3390/ijms22126532 34207103 PMC8234871

[58] Marmolejo DH Wong MYZ Bajalica-Lagercrantz S Tischkowitz M Balmaña J e. E.-G. T. G. 3. Overview of hereditary breast and ovarian cancer (HBOC) guidelines across Europe. Eur J Med Genet (2021) 64:104350. doi: 10.1016/j.ejmg.2021.104350 34606975

[59] Castillo-Guardiola V Rosado-Jimenez L Sarabia-Meseguer MD Marín-Vera M Macías-Cerrolaza JA García-Hernandez R. Next step in molecular genetics of hereditary breast/ovarian cancer: Multigene panel testing in clinical actionably genes and prioritization algorithms in the study of variants of uncertain significance. Eur J Med Genet (2022) 65:104468. doi: 10.1016/j.ejmg.2022.104468 35245693

[60] Ansari FZE Jouali F Marchoudi N Bennani MM Ghailani NN Barakat A. Screening of BRCA1/2 genes mutations and copy number variations in patients with high risk for hereditary breast and ovarian cancer syndrome (HBOC). BMC Cancer (2020) 20:747. doi: 10.1186/s12885-020-07250-0 32778078 PMC7418307

[61] Kumar KR Cowley MJ Davis RL. Next-generation sequencing and emerging technologies. Semin Thromb Hemost (2019) 45:661–73. doi: 10.1055/s-0039-1688446 31096307

[62] Punzón-Jiménez P Lago V Domingo S Simón C Mas A. Molecular management of high-grade serous ovarian carcinoma. Int J Mol Sci (2022) 23:13777. doi: 10.3390/ijms232213777 36430255 PMC9692799

[63] Chougule A Jagtap V Nikam A Kale S Nambiar K Bagayatkar P. Comprehensive development and implementation of good laboratory practice for NGS based targeted panel on solid tumor FFPE tissues in diagnostics. Diagnostics (2020) 12:1291. doi: 10.3390/diagnostics12051291 PMC9141409 35626446

[64] Cazzato G Caporusso C Arezzo F Cimmino A Colagrande A Loizzi V. ForMalin-fixed and paraffin-embedded samples for next generation sequencing: problems and solutions. Genes (2021) 12:1472. doi: 10.3390/genes12101472 34680867 PMC8535326

[65] Asante D-B Calapre L Ziman M Meniawy TM Gray ES. Liquid biopsy in ovarian cancer using circulating tumor DNA and cells: Ready for prime time? Cancer Lett (2020) 468:59–71. doi: 10.1016/j.canlet.2019.10.014 31610267

[66] Zhu JW Charkhchi P Akbari MR. Potential clinical utility of liquid biopsies in ovarian cancer. Mol Cancer (2022) 21:114. doi: 10.1186/s12943-022-01588-8 35545786 PMC9092780

[67] Liany H Lin Y Jeyasekharan A Rajan V. Mining pathway associations from networks of mutual exclusivity interactions. bioRxiv (2020). doi: 10.1101/2020.02.20.957241

[68] Setton J Zinda M Riaz N Durocher D Zimmermann M Koehler M. Synthetic lethality in cancer therapeutics: the next generation. Cancer Discov (2021) 11:1626–35. doi: 10.1158/2159-8290.CD-20-1503 PMC8295179 33795234

[69] da Costa AABA Baiocchi G. Genomic profiling of platinum-resistant ovarian cancer: The road into druggable targets. Semin Cancer Biol (2021) 77:29–41. doi: 10.1016/j.semcancer.2020.10.016 33161141

[70] Gulhan DC Lee JJ-K Melloni GEM Cortés-Ciriano I Park PJ. Detecting the mutational signature of homologous recombination deficiency in clinical samples. Nat Genet (2019) 51:912–9. doi: 10.1038/s41588-019-0390-2 30988514

[71] Schouten PC Richters L Vis DJ Kommoss S van Dijk E Ernst C. Ovarian cancer–specific BRCA-like copy-number aberration classifiers detect mutations associated with homologous recombination deficiency in the AGO-TR1 trial. Clin Cancer Res (2021) 27:6559–69. doi: 10.1158/1078-0432.CCR-21-1673 PMC9401539 34593530

[72] Li Y Nie Y Guo H Guo H Ha C Li Y. Establish of an initial platinum- resistance predictor in high-grade serous ovarian cancer patients regardless of homologous recombination deficiency status. Front Oncol (2022) 12:847085. doi: 10.3389/fonc.2022.847085 35372049 PMC8971787

[73] Lino-Silva LS. Ovarian carcinoma: pathology review with an emphasis in their molecular characteristics. Chin Clin Oncol (2020) 9:45. doi: 10.21037/cco-20-31 32434347

[74] Duncavage EJ Coleman JF de Baca ME Kadri S Leon A Routbort M. Recommendations for the use of in silico approaches for next-generation sequencing bioinformatic pipeline validation. A joint report of the association for molecular pathology, association for pathology informatics, and college of american pathologists. J Mol Diagn (2023) 25:3–16. doi: 10.1016/j.jmoldx.2022.09.007 36244574

[75] Creeden JF Nanavaty NS Einloth KR Gillman CE Stanbery L Hamouda DM. Homologous recombination proficiency in ovarian and breast cancer patients. BMC Cancer (2021) 21:1154. doi: 10.1186/s12885-021-08863-9 34711195 PMC8555001

[76] Li MM Datto M Duncavage EJ Kulkarni S Lindeman NI Roy S. Standards and guidelines for the interpretation and reporting of sequence variants in cancer. A joint consensus recommendation of the association for molecular pathology, american society of clinical oncology, and college of american pathologists. J Mol Diagn (2017) 19:4–23. doi: 10.1016/j.jmoldx.2016.10.002 PMC5707196 27993330

[77] Golubeva VA Nepomuceno TC Monteiro ANA. Germline missense variants in BRCA1: new trends and challenges for clinical annotation. Cancers (2019) 11, 522. doi: 10.3390/cancers11040522 31013702 PMC6520942

[78] Hirotsu Y Schmidt-Edelkraut U Nakagomi H Sakamoto I Hartenfeller M Narang R. Consolidated BRCA1/2 variant interpretation by MH BRCA correlates with predicted PARP inhibitor efficacy association by MH guide. Int J Mol Sci (2020) 21:3895. doi: 10.3390/ijms21113895 32486089 PMC7312854

